# The association positive and negative empathy have with depressive symptoms, resilience, and posttraumatic growth

**DOI:** 10.1038/s41598-025-86285-4

**Published:** 2025-03-19

**Authors:** Taylor Elam, Amber Efthemiou, Kanako Taku

**Affiliations:** Department of Psychology, Oakland University, 654 Pioneer Drive, 205 Pryale Hall, Rochester, MI 48309-4482 USA

**Keywords:** Positive and negative empathy, Trauma, Depressive symptoms, Resilience, Posttraumatic growth, Personality, Psychology, Human behaviour

## Abstract

Empathy, the ability to understand and respond to others’ emotional experiences, is often regarded as a universally positive trait. However, its role in psychological adjustment following adversity is more complex. The current study examined the relationships between empathy – measured globally and through its positive (compassionate concern) and negative (callousness) dimensions – and three outcomes of stress or trauma: depressive symptoms, resilience, and posttraumatic growth (PTG). College students (*N* = 403) completed online surveys assessing these variables, with controls for age, sex, and personality traits. Hierarchical regression analyses showed that global and positive empathy were positively associated with PTG, indicating empathy’s role in fostering personal and relational growth. However, positive empathy also predicted depressive symptoms, reflecting its potential to heighten vulnerability to emotional distress. Negative empathy was inversely related to PTG but unrelated to depressive symptoms or resilience. Resilience demonstrated weaker links with empathy, instead aligning more closely with personality traits like extraversion and conscientiousness. These findings highlight empathy’s dual impact, where it can contribute to personal growth while also increasing susceptibility to distress. Future research should explore empathy’s cognitive and affective components and develop strategies to minimize its negative effects while enhancing adaptive outcomes like PTG.

## Introduction

Empathy, the ability to perceive, understand, and respond sensitively to the emotional experiences of others, is a cornerstone of human interaction^1,2^. By fostering connections, empathy enables individuals to build meaningful relationships, engage in prosocial behaviors^3,4^, and reduces prejudice^5,6^. Empathy is also associated with enhanced life satisfaction^7^, psychological well-being^8^, and interpersonal success^9,10^. Its benefits extend beyond the individual to broader communities, particularly in professional fields such as healthcare where empathy improves patient outcomes and adherence to treatment plans^11,12^. With these findings, empathy has been established as a universally valued trait, often viewed as inherently positive and desirable. However, empathy’s multidimensionality may reveal that it is not uniformly beneficial.

Empathy is a multidimensional construct encompassing cognitive empathy (i.e., understanding the psychological and emotional states of others), affective empathy (i.e., experiencing the emotional states of others), and motor empathy (i.e., synchronizing body language to mirror/match others;^13^). Due to its multidimensional nature, empathy can have the potential to harm as well as heal^14,15^ because while it often promotes positive outcomes such as social bonding and prosocial behavior, it can also lead to empathic distress, emotional exhaustion, and even increased vulnerability to psychiatric conditions like depression for the empathizer^16–18^. Being both harmful and helpful has led to the coinage of empathy as a “double-edged sword”^14,15^. To better understand empathy’s complex nature, Chiorri^19^ proposed a two-factor model using the Toronto Empathy Questionnaire (TEQ;^20^). This model separates overall, or global empathy as a construct into two distinct components: *positive empathy*, derived from statements reflecting compassionate concern and care, and *negative empathy*, derived from statements associated with callousness or empathic distress. Chiorri’s findings^19^ suggest that these two factors (i.e., positive and negative empathy) be measured independently, as they are not simply opposite ends of a spectrum, but distinct constructs with a moderate negative correlation (i.e., correlation coefficient ranging between − 0.22 and − 0.34 throughout a series of four studies;^19^). While global empathy scores provide an overall measure, they may obscure the unique roles of positive and negative empathy when these separate dimensions are not independently assessed as well. By distinguishing between these components, researchers may gain a more comprehensive perspective of empathy’s impact on psychological adjustment outcomes following adversity.

Differentiating between positive and negative empathy may be particularly important when examining its associations with psychological outcomes such as depressive symptoms, resilience, and posttraumatic growth. For example, negative empathy could explain the empathic distress that contributes to depressive symptoms where individuals feel overwhelmed by others’ suffering but powerless to provide relief^21,22^. While in contrast, positive empathy could aid in understanding motivations for prosocial and compassionate engagement with others that helps to foster adaptive psychological outcomes such as resilience and posttraumatic growth (or PTG; the psychological growth one might experience after facing significant adversity;^23,24^). Research suggests that empathy fosters the relational and reflective processes critical to PTG, such as increased compassion and reevaluation of life priorities^23,24^, so the more empathic someone is, the more likely they are to experience PTG. However, PTG often coexists with depressive symptoms, as rumination, core belief disruption, and experiencing some psychological struggle are often prerequisites for personal growth following traumatic life experiences according to the PTG theoretical model^24,25^.

In contrast, resilience is often seen as a protective factor against stress and depression, defined as the ability to recover from adversity^26,27^. However the relationship between resilience and empathy remains complex. While some research suggests that affective empathy might hinder resilience potentially by increasing emotional burden^28^, others indicate that cognitive empathy may enhance resilience through emotional regulation and cognitive flexibility^29,30^. One explanation for these mixed findings is the influence of personality traits, which have been shown to play a significant role in psychological adjustment. One study^31^, for instance, that examined the association between the Big Five personality traits and resilience found that a large percentage of variance within resiliency were explained by personality (i.e., resilience was significantly related to higher levels of openness, conscientiousness, extraversion, agreeableness, and lower levels of neuroticism/emotional stability). Empathy, however, is also positively associated with agreeableness, extraversion, and conscientiousness^32,33^. Therefore, personality may shape how individuals engage with or express empathic experiences, emphasizing a need to consider these traits as potential confounding variables when examining empathy’s effects on psychological outcomes. Despite this, little research has explicitly controlled for personality when investigating the relationships between empathy, depressive symptoms, resilience, and PTG.

Additionally, much of the existing empathy research relies on global measures of the construct despite its growing recognition as multidimensional^34,35^. Thus, further research is needed to adequately capture the distinct contributions of positive and negative empathy in association with psychological adjustment outcomes. The relationships empathy has with depressive symptoms, resilience, and PTG, may be better understood by re-examining empathy as a two-factor structure (i.e., positive empathy and negative empathy). While research traditionally focuses on empathy’s cognitive and affective components, the positive and negative dimensions introduced by the two-factor model may shed new light on conflicting findings within the literature.

## Current study

Taken together, the current study focuses on three ways individuals may react and adapt (i.e., depressive symptoms, resilience, and PTG) after they face adverse experiences in their lives and examines their respective relationships with empathy, measured globally and through its positive and negative dimensions to shed light on its multifunctional nature. Specifically, the current study investigated the relationships empathy has with depressive symptoms, resilience, and PTG after controlling for their known predictors (i.e., demographics and personality characteristics). We hypothesized that empathy would positively be associated with depressive symptoms and PTG, but not resiliency. We also explored the relationships positive empathy and negative empathy (i.e., callousness) have with depressive symptoms, resilience, and PTG to provide novel insights and address critical gaps in the literature. Also, given the known impact of personality traits (i.e., honesty-humility, emotionality, extraversion, agreeableness, conscientiousness, and openness to experience) on psychological processes such as resilience and empathy, we sought to explore the potential associations they may exhibit within the current study.

### Method

#### Participants and procedure

The sample consisted of 403 undergraduate students at a midwestern university in the US with a mean age of 20.22 (*SD* = 3.92, range = 18–50). The majority were female (78.2%). Approximately 53.1% identified as White, 15.1% as African American, 13.9% being of Middle Eastern heritage or descent, 10.4% as Asian, and 7.5% identified as mixed race or other. Additionally, 8.2% of the sample reported being of Hispanic, Latino, or Spanish origin.

Participants were recruited through a university’s subject pool and received course credit upon completion. All participants completed the consent form and then responded anonymously to a 30-minute online survey with questionnaires assessing their demographics, empathy, personality, depressive symptoms, resiliency, traumatic experiences, and PTG. After the participants provided their age, the presentation order of the measures was counterbalanced to avoid any order effects. All procedures were performed in accordance with the principles stated in the Declaration of Helsinki and approved by the university’s institutional review board (IRB-FY2023-215). Inclusion criteria included being 18 years of age or older and completing more than 75% of the survey. Data were analyzed using SPSS 28.

### Measures

#### Empathy

The Toronto Empathy Questionnaire (TEQ;^20^; = 0.86) was used to measure empathy. The TEQ is a commonly used measure assessing global empathy (e.g., emotional comprehension and sympathetic physiological arousal). The participants rated the frequency with which they agreed with 16 statements reflecting various attributes of empathy (e.g., “*When someone else is feeling excited*,* I tend to get excited too*”) or reverse-scored items indicating reduced empathetic concern, (e.g., “*Other people’s misfortunes do not disturb me a great deal*”) on a scale ranging from 0, “*never*,” to 4, “*always*.” While traditionally used to derive a total score indicating global empathy, more recent research (e.g., Chiorri, 2016) has identified a two-factor structure within the TEQ. Specifically, positively worded items reflect positive empathy (e.g., compassionate concern and emotional responsiveness), while reverse-scored items reflect negative empathy or callousness (e.g., lack of empathetic distress). In the current study, both the global empathy score and scores for the two distinct factors, positive empathy and negative empathy, were calculated. Total scores were used with higher scores for overall empathy indicating greater levels of global empathy. Whereas higher scores on the positive empathy factor indicate greater empathetic concern and higher scores on the negative empathy factor indicate greater levels of callousness.

#### Personality

The 60-item HEXACO Personality Inventory (HEXACO-60;^36^) was used to assess the six dimensions of the personality traits: Honesty-Humility, Emotionality, Extraversion, Agreeableness, Conscientiousness, and Openness to Experience. The HEXACO-60 contains 10 items per dimension that were rated on a 5-point Likert scale ranging from 1, “*strongly disagree*,” to 5, “*strongly agree*.” Mean scores for each dimension were calculated. Cronbach’s alpha ranges from 0.71 (Honesty-Humility) to 0.80 (Extraversion).

#### Depressive symptoms

The Patient Health Questionnaire-9 was used to assess depressive symptoms (PHQ-9;^37^; α = 0.89). PHQ-9 is a widely used 9-item measure of depressive symptoms and severity. Participants reported how often in the past two weeks they have been bothered by the following problems (e.g., “*little interest or pleasure in doing things*” or “*feeling tired or having little energy*”). The items are scored on a scale ranging from 0, “*not at all*,” to 3, “*nearly every day*.” Higher total scores indicate greater depressive symptomology.

#### Resiliency

The Brief Resilience Scale (BRS;^38^; = 0.82) which consists of 6 items (e.g., “*I tend to bounce back quickly after hard times*”) was rated on a 5-point Likert scale ranging from 1, “*strongly disagree*,” to 5, “*strongly agree*.” Mean scores were calculated with higher scores indicating higher levels of resiliency.

#### Traumatic events

Participants completed a trauma checklist where they indicated which out of 14 potentially traumatic events (e.g., “natural disaster,” “death of a loved one,” “COVID-19,” etc.) they had experienced in the last five years and identified which event impacted them the most^39,40^.

#### Posttraumatic growth

The PTG Inventory-Short Form (PTGI-SF;^41^; = 0.89) was used to measure the participants’ level of perceived PTG as a result of the traumatic event that most impacted them (e.g., “*I changed my priorities about what is important in life*”). For 10 items, the participants indicated the degree to which each change had occurred for them on a 6-point Likert scale ranging from 0, “*not at all*,” to 5, “*very great degree*.” Mean scores were calculated. Participants that did not identify a single most impactful trauma event (*n* = 49) were excluded when analyzing the data using PTGI-SF scores.

## Results

Overall, participants (*N* = 403) demonstrated similar levels of global empathy (*n*_male_*=* 85, *M*_male_ = 44.32, *SD*_male_ = 7.51; *n*_female_*=* 302, *M*_female_ = 47.59, *SD*_female_ = 9.24) that has been reported in literature (e.g., *M*_male_ = 43.46–44.45; *M*_female_ = 44.62–48.93;^20^). Participants also reported positive empathy scores (*M* = 23.34, *SD* = 5.39) that fell within the mid-range of the TEQ’s possible score distribution, and negative empathy scores (*M* = 8.33, *SD* = 5.06) that were closer to the lower end of the scale. Additionally, participants reported depressive symptom scores (*M* = 8.52, *SD* = 6.13) that were low relative to the maximum possible score of 27 on the PHQ-9. PTG scores (*M* = 3.37, *SD* = 1.05) and resilience scores (*M* = 3.14, *SD* = 0.74) fell in the mid-range of their respective scales, reflecting moderate levels of these outcomes.

As shown in Table 1, positive correlations were found between global empathy and honesty-humility (*r* = .35, *p* = .01), emotionality (*r* = .38, *p* = .01), extraversion (*r* = .16, *p* = .01), agreeableness (*r* = .30, *p* = .01), conscientiousness (*r* = .38, *p* = .01), openness to experience (*r* = .20, *p* = .01), and PTG (*r* = .38, *p* < .01). Similar to previous research studies^19^, positive empathy and negative empathy shared a significant moderate negative association (*r* = − .39, *p* < .001). Positive empathy was positively significantly associated with honesty-humility (*r* = .28, *p* < .01), emotionality (*r* = .34, *p* < .01), extraversion (*r* = .22, *p* < .01), agreeableness (*r* = .25, *p* < .01), conscientiousness (*r* = .34, *p* < .01), openness to experience (*r* = .20, *p* < .01), and PTG (*r* = .43, *p* < .01). On the other hand, negative empathy was negatively significantly associated with honesty-humility (*r* = − .32, *p* < .01), emotionality (*r* = − .29, *p* < .01), agreeableness (*r* = − .26, *p* < .01), conscientiousness (*r* = − .30, *p* < .01), openness to experience (*r* = − .14, *p* = .01), and PTG (*r* = − .22, *p* < .01).

**Table 1:** Means, standard deviations, and correlations among study variables.

	M	SD	Range	1	2	3	4	5	6	7	8	9	10	11	12	13	14
1. Age	20.22	3.92	18–50	−													
2. Sex			1–2	− 0.18***	−												
3. Honesty-humility	3.31	0.62	1–5	0.13*	0.11*	−											
4. Emotionality	3.43	0.64	1–5	− 0.12*	0.40**	0.09	−										
5. Extraversion	3.09	0.58	1–5	0.07	− 0.11*	− 0.08	− 0.14**	−									
6. Agreeableness	3.15	0.58	1– 5	0.00	− 0.07	0.33**	− 0.04	0.13*	−								
7. Conscientiousness	3.53	0.57	1–5	0.04	0.11*	0.32**	0.11*	0.15*	0.13**	−							
8. Openness	3.23	0.61	1–5	0.07	0.04	0.20**	− 0.01	0.04	0.11*	0.22**	−						
9. Global empathy	46.87	8.98	0–64	0.07	0.15**	0.35**	0.38**	0.16**	0.30**	0.38**	0.20**	−					
10. Positive empathy	23.34	5.39	0–32	0.07	0.15**	0.28***	0.34***	0.22***	0.25***	0.34***	0.20***	0.85***	−				
11. Negative empathy	8.33	5.06	0–32	− 0.06	− 0.13*	− 0.32***	− 0.29***	− 0.03	− 0.26***	− 0.30***	− 0.14**	− 0.79***	− 0.39***	−			
12. Depression	8.52	6.13	0–27	− 0.06	0.15**	− 0.08	0.24**	− 0.40**	− 0.13*	− 0.11*	0.08	0.07	0.08	− 0.05	−		
13. Resilience	3.14	0.74	1–5	0.08	− 0.18**	0.03	− 0.49**	0.41**	0.10	0.18**	0.02	0.01	0.04	0.02	− 0.27**	−	
14. PTG	3.37	1.05	0–5	0.02	0.08	0.10	0.06	0.31**	0.20**	0.23**	0.09	0.38**	0.43***	− 0.22***	− 0.06	0.12*	−

Sex was dummy coded as 0 = “Male” and 1 = “Female”. *p < .05, **p < .01, ***p < .001.

### Impact of global empathy

A series of hierarchical regression analyses were then performed to explain the variances in depressive symptoms, resilience, and PTG, with Step 1 including age, sex (0 = male, 1 = female), and 6 dimensions of personality (i.e., honesty-humility, emotionality, extraversion, agreeableness, conscientiousness, and openness to experience), and Step 2 including global empathy.

The results for depressive symptoms (Table 2) found the overall model was significant, *F*(9, 337) = 12.54, *p* < .01, with an adjusted R^2^ = 0.23. The initial model was also significant, *F*(8, 338) = 12.91, *p* < .01, with an adjusted R^2^ = 0.22. When global empathy was added in Step 2, the model was significantly improved (R^2^ change = 0.02, *p* < .01). In addition to the negative associations that honesty-humility (β = − 0.16, *p* < .01) and extraversion (β = − 0.40, *p* < .001) had with depressive symptoms, openness (β = 0.10, *p* < .05) and global empathy (β = 0.17, *p* < .01) showed positive contribution, indicating that higher levels of overall empathy were associated with higher levels of depressive symptoms, after controlling for the demographics and personality traits.

**Table 2:** Hierarchical regression model explaining depressive symptoms assessing the impact of global empathy.

Model	Predictors	B	SE	Beta	CI	F	Adjusted *R*^2^
1	Age	0.02	0.08	0.01	[− 0.13, 0.18]		
	Sex	1.33	0.76	0.09	[− 0.17, 20.83]		
	Honesty-humility	− 1.22	0.55	− 0.12*	[− 2.30, − 0.14]		
	Emotionality	1.45	0.51	0.15**	[0.44, 20.45]		
	Extraversion	− 3.60	0.48	− 0.38***	[− 4.55, − 2.65]		
	Agreeableness	− 0.32	0.53	− 0.03	[− 1.37, 0.73]		
	Conscientiousness	− 0.64	0.57	− 0.06	[− 1.76, 0.48]		
	Openness	1.24	0.50	0.12*	[0.27, 2.22]	12.91***	0.22
2	Age	0.01	0.08	0.01	[− 0.15, 0.17]		
	Sex	1.33	0.76	0.09	[− 0.16, 2.81]		
	Honesty-humility	− 1.54	0.56	− 0.16**	[− 2.63, − 0.45]		
	Emotionality	0.75	0.57	0.08	[− 0.36, 1.86]		
	Extraversion	− 3.86	0.49	− 0.40***	[− 4.82, − 2.90]		
	Agreeableness	− 0.69	0.55	− 0.07	[− 1.76, 0.39]		
	Conscientiousness	− 1.00	0.58	− 0.09	[− 2.14, 0.14]		
	Openness	1.05	0.50	0.10*	[0.07, 2.02]		
	Global empathy	0.12	0.04	0.17**	[0.04, 0.21]	12.54**	0.23

*B* = unstandardized regression coefficient; *SE* standard error, *CI* confidence interval. *p < .05, **p < .01, ***p < .001.

The results for resiliency (Table 3) found the overall model was significant, *F*(9, 341) = 24.49, *p* < .01, with an adjusted R^2^ = 0.38. The initial model was also significant, *F*(8, 342) = 26.88, *p* < .01, with an adjusted R^2^ = 0.37. In Step 1, emotionality negatively contributed (β = − 0.47, *p* < .01), and extraversion (β = 0.29, *p* < .01) and conscientiousness (β = 0.22, *p* < .01) positively contributed. When global empathy was added in Step 2, the model was not significantly improved (R^2^ change = 0.01, *p* = .06), indicating that overall empathy did not make additional contribution toward explaining resiliency after controlling for the demographics and personality traits.

**Table 3:** Hierarchical regression model explaining resilience assessing the impact of global empathy.

Model	Predictors	B	SE	Beta	CI	F	Adjusted *R*^2^
1	Age	0.00	0.01	0.01	[− 0.01, 0.02]		
	Sex	0.06	0.08	0.04	[− 0.10, 0.22]		
	Honesty-humility	0.03	0.06	0.03	[− 0.08, 0.14]		
	Emotionality	− 0.54	0.05	− 0.47***	[− 0.64, − 0.43]		
	Extraversion	0.33	0.05	0.29***	[0.23, 0.43]		
	Agreeableness	− 0.01	0.06	− 0.01	[− 0.12, 0.11]		
	Conscientiousness	0.28	0.06	0.22***	[0.16, 0.39]		
	Openness	− 0.09	0.05	− 0.07	[− 0.19, 0.02]	26.88***	0.37
2	Age	0.00	0.01	0.01	[− 0.02, 0.02]		
	Sex	0.06	0.08	0.04	[− 0.10, 0.22]		
	Honesty-humility	0.01	0.06	0.01	[− 0.10, 0.13]		
	Emotionality	− 0.58	0.06	− 0.51***	[− 0.70, − 0.47]		
	Extraversion	0.31	0.05	0.27***	[0.21, 0.41]		
	Agreeableness	− 0.03	0.06	− 0.03	[− 0.15, 0.08]		
	Conscientiousness	0.25	0.06	0.20***	[0.13, 0.37]		
	Openness	− 0.10	0.05	− 0.08	[− 0.21, 0.01]		
	Global empathy	0.01	0.00	0.10	[0.00, 0.02]	24.49***	0.38

B = unstandardized regression coefficient; *SE* standard error, *CI* confidence interval. *p < 0 0.05, **p < 0 0.01, ***p < 0 0.001.

The results for PTG (Table 4) found that the overall model was significant, *F*(9, 300) = 8.81, *p* < .001, with an adjusted R^2^ = 0.19. The initial model was also significant, *F*(8, 301) = 6.62, *p* < .001, with an adjusted R^2^ = 0.13. In Step 1, extraversion (β = 0.27, *p* < .01), agreeableness (β = 0.12, *p* = .04) and conscientiousness (β = 0.15, *p* < .01), were positively associated with PTG. When global empathy was added in Step 2, the model was significantly improved (R^2^ change = 0.06, *p* < .01). In addition to extraversion (β = 0.22, *p* < .001), global empathy was significant (β = 0.32, *p* < .001), indicating that, after controlling for the personality traits and demographics, higher levels of overall empathy were associated with higher levels of PTG.

**Table 4:** Hierarchical regression model explaining PTG assessing the impact of global empathy.

Model	Predictors	B	SE	Beta	CI	F	Adjusted *R*^2^
1	Age	0.00	0.02	0.01	[− 0.03, 0.03]		
	Sex	0.13	0.15	0.05	[− 0.16, 0.42]		
	Honesty-humility	0.01	0.10	0.01	[− 0.19, 0.22]		
	Emotionality	0.13	0.10	0.08	[− 0.06, 0.32]		
	Extraversion	0.43	0.09	0.27***	[0.25, 0.62]		
	Agreeableness	0.21	0.10	0.12*	[0.01, 0.42]		
	Conscientiousness	0.28	0.11	0.15**	[0.07, 0.49]		
	Openness	− 0.00	0.09	− 0.00	[− 0.19, 0.18]	6.62***	0.13
2	Age	− 0.00	0.01	− 0.01	[− 0.03, 0.03]		
	Sex	0.12	0.14	0.05	[− 0.16, 0.40]		
	Honesty-humility	− 0.07	0.10	− 0.04	[− 0.27, 0.13]		
	Emotionality	− 0.07	0.10	− 0.04	[− 0.27, 0.13]		
	Extraversion	0.35	0.09	0.22***	[0.17, 0.53]		
	Agreeableness	0.10	0.10	0.06	[− 1.10, 0.30]		
	Conscientiousness	0.17	0.11	0.10	[− 0.04, 0.38]		
	Openness	− 0.06	0.09	− 0.03	[− 0.26, 0.12]		
	Global empathy	0.04	0.01	0.32***	[0.02, 0.05]	8.81***	0.19

B unstandardized regression coefficient; *SE* standard error, *CI* confidence interval. *p < .05, **p <0 0.01, ***p <0 0.001.

### Impact of positive and negative empathy

Another series of hierarchical regression analyses were performed to explore the unique contributions of positive and negative empathy in explaining the variances in depressive symptoms, resilience, and PTG, with Step 1 including age, sex (0 = male, 1 = female), and 6 dimensions of personality (i.e., honesty-humility, emotionality, extraversion, agreeableness, conscientiousness, and openness to experience), and Step 2 including positive and negative empathy.

The results for depressive symptoms (Table 5) found the overall model was significant, *F*(10, 336) = 11.72, *p* < .01, with an adjusted R^2^ = 0.24. The initial model was also significant, *F*(8, 338) = 12.91, *p* < .01, with an adjusted R^2^ = 0.22. When positive and negative empathy were added in Step 2, the model was significantly improved (R^2^ change = 0.03, *p* < .01). In addition to the negative associations that honesty-humility (β = − 0.15, *p* < .01) and extraversion (β = − 0.42, *p* < .001) had with depressive symptoms, openness (β = 0.10, *p* < .05) and positive empathy (β = 0.19, *p* < .01) showed positive contribution, indicating that higher levels of positive empathy, but not negative empathy, were associated with higher levels of depressive symptoms, after controlling for the demographics and personality traits.

**Table 5:** Hierarchical regression model explaining depressive symptoms assessing the impact of positive and negative empathy.

Model	Predictors	B	SE	Beta	CI	F	Adjusted *R*^2^
1	Age	0.02	0.08	0.01	[− 0.13, 0.18]		
	Sex	1.33	0.76	0.09	[− 0.17, 2.83]		
	Honesty-humility	− 1.22	0.55	− 0.12*	[− 2.30, − 0.14]		
	Emotionality	1.45	0.51	0.15**	[0.44, 2.45]		
	Extraversion	− 3.60	0.48	− 0.38***	[− 4.55, − 2.65]		
	Agreeableness	− 0.32	0.53	− 0.03	[− 1.37, 0.73]		
	Conscientiousness	− 0.64	0.57	− 0.06	[− 1.76, 0.48]		
	Openness	1.24	0.50	0.12*	[0.27, 2.22]	120.91***	0.22
2	Age	0.01	0.08	0.00	[− 0.15, 0.16]		
	Sex	1.28	0.75	0.09	[− 0.20, 2.76]		
	Honesty-humility	− 1.50	0.55	− 0.15**	[− 2.59, − 0.42]		
	Emotionality	0.69	0.56	0.07	[− 0.42, 1.80]		
	Extraversion	− 4.03	0.49	− 0.42***	[− 5.00, − 3.05]		
	Agreeableness	− 0.68	0.54	− 0.07	[− 1.75, 0.39]		
	Conscientiousness	− 0.98	0.58	− 0.09	[− 2.12, 0.15]		
	Openness	0.99	0.50	0.10*	[0.01, 1.96]		
	Positive empathy	0.22	0.07	0.19**	[0.09, 0.35]	11.72***	0.24
	Negative empathy	− 0.03	0.07	− 0.02	[− 0.16, 0.11]		

*B* = unstandardized regression coefficient; *SE* standard error, *CI* confidence interval. *p < .05, **p < .01, ***p < .001.

The results for resiliency (Table 6) found the overall model was significant, *F*(10, 340) = 21.99, *p* < .01, with an adjusted R^2^ = 0.38. The initial model was also significant, *F*(8, 342) = 26.88, *p* < .01, with an adjusted R^2^ = 0.37. In Step 1, emotionality negatively contributed (β = − 0.47, *p* < .01), and extraversion (β = 0.29, *p* < .01) and conscientiousness (β = 0.22, *p* < .01) positively contributed. When positive and negative empathy were added in Step 2, the model was not significantly improved (R^2^ change = 0.01, *p* = .15), indicating that positive and negative empathy did not make additional contributions toward explaining resiliency after controlling for the demographics and personality traits.

**Table 6:** Hierarchical regression model explaining resilience assessing the impact of positive and negative empathy.

Model	Predictors	B	SE	Beta	CI	F	Adjusted *R*^2^
1	Age	0.00	0.01	0.01	[− 0.01, 0.02]		
	Sex	0.06	0.08	0.04	[− 0.10, 0.22]		
	Honesty-humility	0.03	0.06	0.03	[− 0.08, 0.14]		
	Emotionality	− 0.54	0.05	− 0.47***	[− 0.64, − 0.43]		
	Extraversion	0.33	0.05	0.29***	[0.23, 0.43]		
	Agreeableness	− 0.01	0.06	− 0.01	[− 0.12, 0.11]		
	Conscientiousness	0.28	0.06	0.22***	[0.16, 0.39]		
	Openness	− 0.09	0.05	− 0.07	[− 0.19, 0.02]	26.88***	0.37
2	Age	0.00	0.01	0.01	[− 0.02, 0.02]		
	Sex	0.06	0.08	0.04	[− 0.10, 0.22]		
	Honesty-humility	0.01	0.06	0.01	[− 0.11, 0.13]		
	Emotionality	− 0.58	0.06	− 0.51***	[− 0.70, − 0.47]		
	Extraversion	0.31	0.05	0.27***	[0.21, 0.42]		
	Agreeableness	− 0.03	0.06	− 0.03	[− 0.15, 0.08]		
	Conscientiousness	0.25	0.06	0.20***	[0.13, 0.37]		
	Openness	− 0.10	0.05	− 0.08	[− 0.21, 0.01]		
	Positive Empathy	0.01	0.01	0.07	[− 0.01, 0.02]	21.99***	0.38
	Negative Empathy	− 0.01	0.01	− 0.06	[− 0.02, 0.01]		

B = unstandardized regression coefficient; *SE* standard error, *CI* confidence interval. *p < .05, **p < .01, ***p <0 0.001.

The results for PTG (Table 7) found that the overall model was significant, *F*(10, 299) = 9.55, *p* < .001, with an adjusted R^2^ = 0.22. The initial model was also significant, *F*(8, 301) = 6.62, *p* < .001, with an adjusted R^2^ = 0.13. In Step 1, extraversion (β = 0.27, *p* < .01), agreeableness (β = 0.12, *p* = .04) and conscientiousness (β = 0.15, *p* < .01), were positively associated with PTG. When positive and negative empathy were added in Step 2, the model was significantly improved (R^2^ change = 0.09, *p* < .01). In addition to extraversion (β = 0.18, *p* < .01), positive empathy was significant (β = 0.37, *p* < .001), indicating that, after controlling for the personality traits and demographics, higher levels of positive empathy, but not negative empathy, were associated with higher levels of PTG.

**Table 7:** Hierarchical regression model explaining PTG assessing the impact of positive and negative empathy.

Model	Predictors	B	SE	Beta	CI	F	Adjusted *R*^2^
1	Age	0.00	0.02	0.01	[− 0.03, 0.03]		
	Sex	0.13	0.15	0.05	[− 0.16, 0.42]		
	Honesty-humility	0.01	0.10	0.01	[− 0.19, 0.22]		
	Emotionality	0.13	0.10	0.08	[− 0.06, 0.32]		
	Extraversion	0.43	0.09	0.27***	[0.25, 0.62]		
	Agreeableness	0.21	0.10	0.12*	[0.01, 0.42]		
	Conscientiousness	0.28	0.11	0.15*	[0.07, 0.49]		
	Openness	− 0.00	0.09	− 0.00	[− 0.19, 0.18]	6.62***	0.13
2	Age	− 0.01	0.01	− 0.02	[− 0.03, 0.02]		
	Sex	0.08	0.14	0.03	[− 0.19, 0.36]		
	Honesty-humility	− 0.06	0.10	− 0.04	[− 0.27, 0.13]		
	Emotionality	− 0.07	0.10	− 0.04	[− 0.27, 0.13]		
	Extraversion	0.29	0.09	0.18**	[0.11, 0.47]		
	Agreeableness	0.11	0.10	0.06	[− 0.09, 0.31]		
	Conscientiousness	0.19	0.11	0.10	[− 0.02, 0.39]		
	Openness	− 0.08	0.09	− 0.05	[− 0.25, 0.10]		
	Positive empathy	0.07	0.01	0.37***	[0.05, 0.10]	9.55***	0.22
	Negative empathy	− 0.00	0.01	− 0.00	[− 0.03, 0.03]		

B = unstandardized regression coefficient; *SE* standard error, *CI* confidence interval. *p < .05, **p < .01, ***p < .001.

## Discussion

Stressful and potentially traumatic life experiences can lead to a variety of psychological outcomes, including depressive symptoms, resilience, and posttraumatic growth (PTG). Some individuals may experience heightened emotional distress, manifesting as depressive symptoms, while others demonstrate resilience, recovering quickly from adversity due to intrinsic personal attributes that buffer against negative psychological outcomes. Still, others may experience profound psychological growth, such as PTG, which involves finding new meaning and personal strength in the aftermath of hardship^24^. Empathy, as a multifaceted construct, plays a pivotal role in these processes. Although empathy is traditionally viewed as a beneficial interpersonal skill, it can also heighten vulnerability to emotional exhaustion and psychological disorders such as depression. The dual nature of empathy emphasizes the importance of understanding its distinct dimensions (i.e., positive empathy or compassionate concern and negative empathy or callousness) and their unique relationships with psychological outcomes. The current study expanded upon prior literature by examining the relationships between global, positive, and negative empathy and three psychological adjustment outcomes following adversity: depressive symptoms, resilience, and PTG. Importantly, our investigation accounted for the confounding effects of age, sex, and personality traits by using the HEXACO personality framework.

The strong correlations observed between global and positive empathy and all six HEXACO personality traits highlight the prosocial nature of these dimensions. Traits such as honesty-humility, emotionality, and agreeableness may be more likely to enhance individuals’ capacity for compassionate concern and emotional responsiveness, fostering interpersonal success and psychological growth. On the other hand, negative empathy was negatively associated with honesty-humility, agreeableness, conscientiousness, and openness to experience, possibly reflecting its alignment with less adaptive personality profiles. Our findings reinforce the importance of controlling for personality traits in order to better isolate the unique contributions of empathy on psychological adjustment outcomes.

In support of our hypothesis, global empathy as well as positive empathy were positively associated with PTG, reinforcing the idea that empathy facilitates adaptive processes such as meaning-making, relational growth, and increased compassion following adversity. From a theoretical perspective, these findings align with the PTG framework, which posits that growth often coexists with psychological struggle^24,25^. The significant association between positive empathy and PTG suggests that empathic concern may facilitate the reflective and relational processes necessary for growth, even as it heightens sensitivity to distress. Interestingly, neither global empathy nor positive empathy was significantly related to depressive symptoms in the correlation analyses, however; positive empathy emerged as a significant predictor of depressive symptoms in the regression analyses. Individuals with high levels of positive empathy may be more attuned to the emotional suffering of others, potentially heightening their susceptibility to empathic distress and depressive symptoms under certain conditions^16,21^. Negative empathy, on the other hand, showed distinct relationships with depressive symptoms, resilience, and PTG. While it was inversely related to PTG in the correlations, it did not predict depressive symptoms, resilience, or PTG in the regression models. Perhaps this highlights its unique role as a maladaptive dimension of empathy, potentially impeding growth and relational engagement without directly exacerbating emotional distress. It is possible that negative empathy is more strongly linked to outcomes such as interpersonal conflict or hostility, as suggested by its negative associations with personality traits like agreeableness and conscientiousness.

Moreover, resilience demonstrated weaker and less consistent relationships with empathy compared to depressive symptoms and PTG. Although small positive correlations were observed between global and positive empathy with resilience, these relationships were not significant in the regression analyses, suggesting that resilience may rely more heavily on intrinsic personality traits, such as emotionality, extraversion, and conscientiousness, rather than on empathic engagement with others. The independence of resilience from empathy, and both its positive and negative dimensions, aligns with prior studies emphasizing the intrapersonal nature of resilience, which involves cognitive flexibility, emotion regulation, and a positive outlook rather than interpersonal processes^30,42^.

Taken together, the results of this study provide empirical support for the two-factor structure of empathy and its utility in understanding the complex interplay between empathy and psychological adjustment. By disentangling the contributions of positive and negative empathy, our findings help to highlight the double-edged nature of empathy: while positive empathy promotes relational and psychological growth, it may also increase vulnerability to emotional distress. Negative empathy, on the other hand, appears to undermine adaptive processes without necessarily exacerbating depressive symptoms.

### Implications

The current study sheds light on the multifaceted role of empathy in psychological adjustment, offering valuable knowledge with both clinical and societal implications. A focus on empathy and its relationship with depression and PTG may have direct and paramount influence on the clinical realm of psychology. Positive empathy, with its capacity to promote relational growth and meaning-making, may be a helpful tool in fostering trauma recovery. Clinicians may find it beneficial to incorporate empathy-focused interventions, such as perspective-taking exercises in treatment in order to encourage clients to connect meaningfully with others and foster adaptive growth following adverse events. However, since positive empathy and depressive symptoms can cooccur, interventions must consider a balanced approach for increasing empathy, fostering PTG, while mitigating against depressive symptomatology. For example, for individuals with high levels of affective empathy, therapeutic strategies should emphasize emotional regulation, such as mindfulness or cognitive restructuring, to mitigate empathic distress and reduce vulnerability to emotional exhaustion. Empathy training programs, while beneficial, must be carefully tailored to avoid exacerbating empathic distress, especially in populations already prone to emotional exhaustion. These programs could prioritize enhancing cognitive empathy which promotes understanding without the emotional toll of affective empathy. Such an approach could benefit caregivers, educators, and others in emotionally demanding roles, equipping them to engage compassionately without compromising their well-being. The findings on negative empathy, as measured by its inverse correlation with PTG and certain personality traits (e.g., agreeableness, conscientiousness, honesty-humility), showcase its potential role as a maladaptive dimension of empathy. Unlike positive empathy, which fosters psychological growth and relational engagement, negative empathy may reflect a more detached or callous orientation, limiting the mechanisms that facilitate PTG, such as relational connection or reflective processing and further aligns with prior research suggesting that empathic distress or a lack of compassionate engagement can hinder adaptive outcomes^21,22^. While negative empathy did not emerge as a significant predictor of PTG, depressive symptoms, or resilience in the regression models, its associations with less adaptive personality profiles may indicate its influence in shaping broader interpersonal challenges rather than direct psychological outcomes.

### Limitations

Although the current study offers novel insight to the understanding of empathy and its relationship with depression, resiliency, and PTG; it is not without limitations. The cross-sectional design prevents us from establishing causality, leaving open the possibility that psychological outcomes influence empathy levels rather than the reverse. Longitudinal research is needed to clarify these relationships and explore how empathy evolves following traumatic events and experiences. Measurement limitations also restricted the scope of our findings. The Toronto Empathy Questionnaire (TEQ)^20^ captures global empathy but does not distinguish between cognitive and affective components. Future studies should employ tools that assess these dimensions separately, offering greater insight into how each contributes to depressive symptoms, resilience, and PTG (i.e., cognitive empathy, affective empathy, positive empathy, and negative empathy). Additionally, the sample, composed primarily of American college students, may not be representative of broader populations, limiting the generalizability of our findings. Additionally, relying on self-report measures may introduce potential biases, such as social desirability or response distortion. Incorporating observational or experimental methods could complement self-reported data and strengthen the validity of future research. Unexamined factors, such as gender roles, interpersonal sensitivity, and dark personality traits (e.g., narcissism, Machiavellianism, psychopathy) may have influenced the findings and should also be considered in future studies.

### Future directions

Future research should build on these findings by utilizing measures and scales that separately assess all dimensions of empathy (i.e., positive, negative, cognitive and affective). Such tools could deepen our understanding of the distinct roles these dimensions play in psychological outcomes like depressive symptoms, resilience, and PTG. Longitudinal studies are needed to track changes in empathy over time and examine how it interacts with psychological outcomes in different contexts. Expanding research to include diverse populations is also critical. Investigating empathy’s effects across different cultural contexts, age groups, and marginalized communities could reveal important variations and inform tailored interventions. For example, examining empathy in collectivist versus individualist cultures could highlight how societal values influence its role in psychological adjustment. Similarly, exploring empathy within underrepresented or underserved populations, such as those with limited access to mental health resources, would help to provide more context and understanding of empathy’s role in psychological adjustment outcomes.

Given the potential downsides of excessive empathy, such as empathic distress or emotional exhaustion, future studies should aim to identify optimal levels of empathy for well-being. Research into fostering cognitive empathy without unintentionally increasing affective empathy may be particularly beneficial for individuals prone to emotional sensitivity. Understanding how empathy can be cultivated effectively and safely will be crucial for designing interventions that maximize its benefits while minimizing its risks. Exploring factors such as caregiving roles, workplace environments, or digital interactions would offer valuable insights into how empathy functions across various domains of life. Additionally, exploring a broader range of outcomes, including posttraumatic stress disorder (PTSD), coping mechanisms, and interpersonal dynamics, would provide a more comprehensive understanding of empathy’s role in trauma recovery. The current study’s findings, which emphasize the distinct roles of positive and negative empathy, and their contributions to psychological adjustment, serve as a critical foundation for guiding these explorations.

## Data Availability

The authors confirm that the data supporting the findings of this study are available within the article and tables.
